# Natural antibody responses to *Plasmodium falciparum* MSP3 and GLURP(R0) antigens are associated with low parasite densities in malaria patients living in the Central Region of Ghana

**DOI:** 10.1186/s13071-017-2338-7

**Published:** 2017-08-23

**Authors:** L. E. Amoah, S. V. Nuvor, E. K. Obboh, F. K. Acquah, K. Asare, S. K. Singh, J. N. Boampong, M. Theisen, K. C. Williamson

**Affiliations:** 1 Noguchi Memorial Institute for Medical Research, University of Ghana, Accra, Ghana; 2 Department of Microbiology and Immunology, School of Medical Sciences, University of Cape Coast, Cape Coast, Ghana; 3 School of Medical Sciences University of Cape Coast, Cape Coast, Ghana; 4 Department of Biomedical Sciences University of Cape Coast, Cape Coast, Ghana; 5 Department for Congenital disorders, Statens Serum Institut, Copenhagen, Denmark; 6 Centre for Medical Parasitology, Department of International Health, Immunology and Microbiology, University of Copenhagen, Copenhagen, Denmark; 7 Department of Infectious Diseases, Copenhagen University Hospital, Rigshospitalet, Copenhagen, Denmark; 8 Loyola University Chicago, Chicago, IL USA; 9 Uniform Services University of the Health Sciences, Bethesda, MD USA

**Keywords:** Genetic diversity, Multiplicity of infection, *msp1*, *msp2*, MSP3, GLURP

## Abstract

**Background:**

*Plasmodium falciparum* genetic diversity and multiplicity of infection (MOI) are parasite features that have been suggested to influence the acquisition of protective immunity against malaria. This study sought to assess the relationship between MOI and parasite density (PD) in malaria patients living in the Central Region of Ghana and to determine whether naturally occurring antibody levels against *P. falciparum* GLURP (PF3D7_1035300) and MSP3 (PF3D7_1035400) antigens are associated with decreased parasite load.

**Methods:**

Dried filter paper blood blots were obtained from children and adults diagnosed with uncomplicated *P. falciparum* malaria. Microscopy was used to estimate *P. falciparum* parasite density and polymerase chain reaction (PCR) amplification of the polymorphic regions of *msp1* (PF3D7_0930300) and *msp2* (PF3D7_0206800) was used for parasite genotyping and MOI determination*.* ELISA was used to measure the serum IgG concentration of R0 fragment of GLURP (GLURP(R0)) and MSP3 antibodies.

**Results:**

All 115 samples were positive for *P. falciparum* by PCR using either the *msp1* or *msp2* genotyping primer sets. The most prevalent *msp1* and *msp2* alleles were KI and 3D7, respectively. The geometric mean (GM) for MOI determined by both *msp1* and *msp2* genotyping was 1.3 for the entire population and was generally higher in children than in adults. Seropositivity was estimated at 67 and 63% for GLURP(R0) and MSP3 antibodies, respectively, and antibody titers were negatively correlated with parasite density.

**Conclusions:**

The negative correlation between naturally occurring GLURP(R0) and MSP3 antibody levels and parasite density observed in this study suggest that augmenting the antibody response with the GMZ2 vaccine could enhance protection in the Central Region of Ghana.

**Electronic supplementary material:**

The online version of this article (doi:10.1186/s13071-017-2338-7) contains supplementary material, which is available to authorized users.

## Background

The 2015 annual report of the Ghana National Malaria Control Program reported that 39.2% of all the Outpatient Department cases recorded in the Central Region were due to malaria [1]. This 44.5% increase in reported malaria cases from 2014 to 2015 [1] suggests that malaria still remains a disease of public health concern despite the enormous advances in interventions to control the disease. The enormous genetic diversity found in the *P. falciparum* parasites is likely to complicate disease control. This diversity within *P. falciparum* has served as a key survival mechanism for the parasite, as it allows for immune evasion as well as drug resistance [2]. Major malaria treatment regimens such as chloroquine [3] have already failed and tolerance and resistance to artemisinin combination therapy has been reported in some malaria endemic countries [4, 5]. Specific *P. falciparum* parasite genotypes, such as PfEMP1-DBL1α, have also been implicated with severe malaria [6] and the CAMP(C-) genotype of EBA 175 associated with severe malaria and fatal outcome [7], again suggesting parasite diversity plays an important role in the clinical manifestation and pathology of malaria [8]. Merozoite surface antigens of the parasite such as MSP1 and MSP2 are exposed directly to immune pressure and found to be highly polymorphic. *Plasmodium falciparum* merozoite surface proteins 1 (MSP1: PF3D7_0930300) and 2 (MSP2: PF3D7_0206800) are widely used in the field for determining parasite diversity and multiplicity of infection. The *msp1* gene has been divided into three allelic families namely K1, MAD20, and RO33 type based on the diversity within the Block 2 region of the gene sequence [9]. The *msp2* gene is dimorphic [10] based on diversity with the Block 3 region of its gene sequence and it is classified as being in the FC27 or 3D7 allele family. These merozoite surface antigens have proven to be vital tools in characterizing *P. falciparum* parasites [11].

Infections of *P. falciparum* have also been implicated in the maintenance of strain-specific anti-merozoite antibodies. An increase in the number of FC27 alleles in an infection has been associated with increased antibody titres to the MSP2-Dd2 (FC27 family allele) but not MSP2-Ch150/9 (3D7 family allele) antigen [12]. To cover both 3D7 and FC27 allele families, a MSP1-C1 combination vaccine containing 3D7 and FC27 alleles was developed, but unfortunately it showed unacceptable reactogenicity limiting further analysis [13]. Multiclonal infections, consisting of diverse concurrent clones, could be advantageous to the host as it enables the acquisition of antibodies to genetically diverse parasites (increased breadth of antibodies) as has been shown in asymptomatic infections [11, 14]. It has also been reported to reduce the risk of clinical illness during a subsequent infection with a clonally similar parasite [15, 16], as antibody titers maintained by multiclonal asyptomatic infection may be more likely to arrest the growth of newly acquired, but clonally similar parasites [17].

In addition to merozoite surface proteins, parasite antigens released when mature schizont-infected erythrocytes burst are also exposed to host immune defenses and one of these, GLURP(R0), is included in a current vaccine candidate GMZ2 [18–20]. GMZ2 is a chimeric malaria vaccine composed of the R0 fragment of GLURP(R0) fused to MSP3 [21] and is undergoing vaccine trials in a number of African countries including Ghana [22]. Antibodies against both GLURP(R0) and MSP3 have been implicated in the acquisition of protective immunity to malaria [23]. The present study was designed to identify possible associations between the natural immune response to GLURP(R0) and MSP3, parasite density and multiplicity of infection in the forest zone of the Central Region of Ghana.

## Methods

### Study site and sample acquisition

**Fig. 1:**
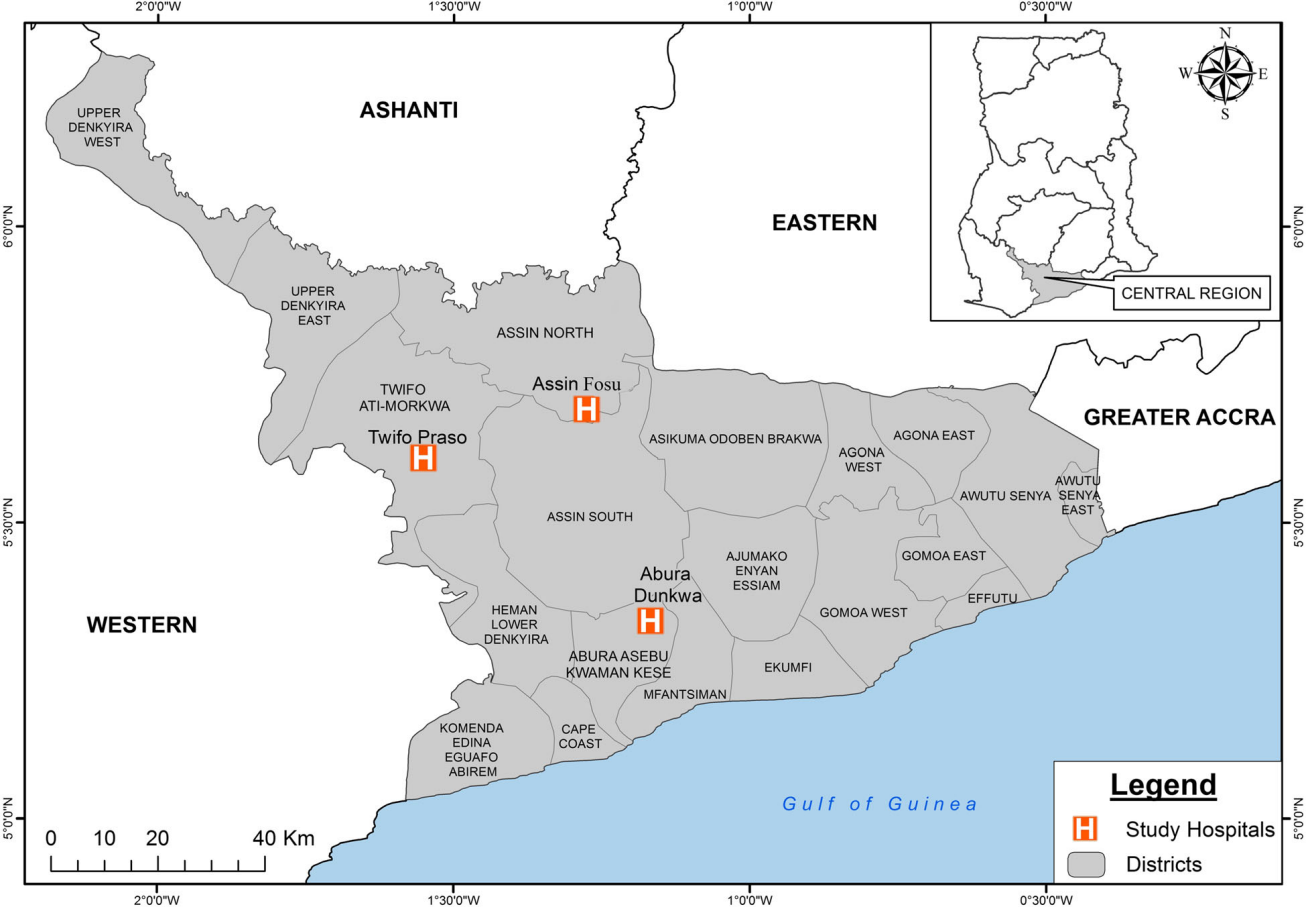
Map of Ghana showing study sites. Samples were collected from the hospitals marked H located in Twifo Praso, Assin Fosu and Abura Dunkwa in the Central Region of Ghana. Twifo Praso is the district capital of Twifo-Ati Morkwa district, Assin Fosu in the district capital of Assin North Municipality and Abura Dunkwa is the district capital of Abura-Asebu Kwaman Kese

Peripheral whole blood samples used for dried filter paper blood blots, thick and thin blood smears and harvesting of serum were collected from each participant as part of a larger study from three districts in the Central Region of Ghana [24]. The hemoglobin concentrations of the blood samples were measured directly using automated blood analyzers in the various study hospitals. The study was conducted a district hospital located in Abura Dunkwa, Twifo Praso and Assin Fosu in the Central Region of Ghana (Fig. 1). All the three study sites are in the forest zone of Ghana and share common peak (April-September) and off peak (November-March) malaria seasons. In 2013, the incidence of clinical malaria ranged between 11 and 15% in the three sites [25, 26].

### Extraction of parasite DNA

Two 3 mm punches (discs) of dried blood blots from the filter paper were used for gDNA extraction using the Saponin-Chelex extraction method [27]. Briefly, discs for each sample were incubated in 1120 μl of a 0.5% saponin/phosphate buffered saline (PBS) solution overnight at room temperature on a shaking incubator. After incubation, the discs were washed twice with 1 ml PBS, followed by centrifugation at 10,000× g. Subsequently, 150 μl of a 6% Chelex-100 (Sigma-Aldrich, Missouri, USA) in DNase/RNase free water was added to the discs and incubated for 5 min at 95 °C. After a final high-speed centrifugation, the supernatant containing the gDNA was either used immediately for the genotyping PCR amplification reactions or stored at - 20 °C.

### Molecular identification and genotyping

To distinguish between the three major *msp1* allelic families (K1, MAD 20, and RO33) and the two *msp2* allelic families (FC27 and 3D7), nested PCR was performed using family-specific primers [28] shown in Table 1. The outer PCR reaction mixture contained 200 nM dNTP, 2 mM MgCl_2_, 133 nM of each forward and reverse primer, 0.5 units of One Taq DNA polymerase (New England BioLab, MA, USA) and 4 μl (about 0.25 μl of whole blood) of genomic DNA (gDNA) template. The inner nested reaction mixture contained 200 nM dNTP, 1.8 mM MgCl_2_, 200 nM of each forward and reverse primer and 0.5 unit of One Taq DNA polymerase supplemented with 0.5 μl of the primary PCR product. The cycling parameters for the nested PCR consisted of an initial denaturation at 94 °C for 3 min, followed by 30 cycles at 94 °C for 1 min; 50–59 °C (optimized for each primer pair) for 35 s, and 68 °C for 2 min 30 s; with a final extension at 68 °C for 5 min. The PCR reactions were all run on a Biometra TAdvance thermal cycler (Göttingen, Germany). Positive gDNA controls for each allelic family (3D7 strain - alleles, *msp2* 3D7/*msp1* KI; K1 strain - alleles, *msp1* KI/*msp2* FC27; HB3 strain - alleles *msp1* MAD20/*msp2* FC27 and RO33 strain - alleles, *msp1* RO33/*msp2* 3D7 were from MR4) and no template negative control were included in each set of PCR reactions. PCR products were separated on 2% ethidium bromide-stained agarose gels and visualized under UV illumination.

**Table 1:** *msp1* and *msp2* genotyping primers

Marker	Primer name	Primer sequence (5′-3′)	Annealing temperature (°C)
*msp1*			
Primary	M1- OF	CTAGAAGCTTTAGAAGATGCAGTATTG	54
	M1- OR	CTTAAATAGTATTCTAATTCAAGTGGATCA	
K1	M1- KF	AAATGAAGAAGAAATTACTACAAAAGGTGC	59
	M1- KR	GCTTGCATCAGCTGGAGGGCTTGCACCAGA	
MAD 20	M1- MF	AAATGAAGGAACAAGTGGAACAGCTGTTAC	59
	M1- MR	ATCTGAAGGATTTGTACGTCTTGAATTACC	
RO33	M1- RF	TAAAGGATGGAGCAAATACTCAAGTTGTTG	59
	MI- RR	CAAGTAATTTTGAACTCATGTTTTAAATCAGCGTA	
*msp2*			
Primary	M2- OF	ATGAAGGTAATTAAAACATTGTCTATTATA	54
	M2- OR	CTTTGTTACCATCGGTACATTCTT	
3D7/FC27- F	S1fw	GCTTATAATATGAGTATAAGGAGAA	50
FC27- R	M5rev	GCATTGCCAGAACTTGAA	
3D7 - R	N5rev	CTGAAGAGGTACTGGTAGA	

### Multiplicity of infection

The number of concurrent infecting genotypes contained in each sample, which represents the multiplicity of infection (MOI) for that sample, was calculated as the highest number of alleles obtained in the sample after either *msp1* or *msp2* genotype reactions. Samples with more than one PCR fragment per marker (infecting genotype) were considered as containing multiclonal infections, whereas samples with only one infecting genotype for either marker were considered as clonal with respect to that marker. The geometric mean (GM) of MOI for both *msp1* and *msp2* genotyping was calculated by determining the GM for the sum of the MOI identified for each sample divided by the total number of samples positive for the same marker.

### GLURP(R0) and MSP3 ELISA

One hundred microliters of affinity purified GLURP(R0) [19, 29] or MSP3 [19, 29] in PBS at 1 μg/ml was used to coat 80 wells of a NUNC maxisorp 96-well ELISA plate overnight at 4 °C. The remaining 16 wells were filled with 100 μl of purified human polyclonal IgG (PB055, The Binding Site) serially titrated to serve as standards. The plates were subsequently washed four times with 1× PBS with 0.05% Tween 20 (1× PBST), blocked with 150 μl of 3% skimmed milk in 1× PBST, washed twice and incubated for 1 h with 100 μl of test serum or a pool of negative control serum from donors living in non-endemic countries, diluted 1:200 in 1% skimmed milk in 1× PBST. Subsequently, after four 1× PBST washes, bound IgG were probed with polyclonal rabbit anti-human IgG-horseradish peroxidase (1:3000 dilution in 1% skimmed milk in 1× PBST) for 1 h. Plates were then washed four times as described above and the bound conjugate detected by incubating with 50 μl of tetramethylbenzidine substrate and stopping the reaction with 50 μl of 0.2 M sulphuric acid. ELx808 Absorbance Reader (BioTek, VT, USA) was used to read the ODs of each sample.

### Data analysis

Data were entered into Excel, converted to concentrations using ADAMSEL (Ed Remarque) and analyzed using GraphPad Prism v7 and SPSS v.22 (IBM). The frequency of each *msp1* and *msp2* allelic family as well as the geometric mean and corresponding 95% confidence interval were calculated using frequency distribution in GraphPad Prism. The correlation between age and PD, GLURP(R0) and MSP3 IgG concentrations was performed using GraphPad Prism. SPSS descriptive statistics (cross tabs) was used to determine the descriptive statistics for the participants per study site (Additional file 1: Table S1). SPSS nonparametric tests (independent samples) were used to determine the association between IgG concentrations and antibody seropositivity with MOI and parasite density and also to draw graphs showing the relationship between PD, Age and IgG seropositivity (Additional file 2: Figure S1). Statistical significance was defined as *P* value ≤ 0.05 unless otherwise stated. Using an estimate of 8000 white blood cells/μl of blood, the number of asexual blood stage parasites was determined for 200 WBCs and multiplied by 40.

## Results

The study population included 115 symptomatic malaria patients, 47 (41%) were from Assin Fosu, 36 (31%) from Twifo Praso and 32 (28%) from Abura Dunkwa (Fig. 1). The geometric mean (GM) age of the study population was 6.37 (95% CI: 5.1–7.97) years. The GM of the *P. falciparum* parasite density (PD) was 7715 (95% CI: 5719–10,408) parasites/μl blood with a standard error of the mean of 3230. The GM of the hemoglobin concentrations within the population was 9.4 (95% CI: 9.0–9.8) (Table 2). Due to the similarities in demographic features between the three study sites (Additional file 1: Table S1), data analysis was performed on the total sample set without differentiating according to study site. Although children had relatively higher parasite densities than the adults, the correlation was not significant (Spearman’s *r* = -0.949, *P* = 0.35; Additional file 3: Figure S3).

**Table 2:** Background characteristics of study participants

	Age (years)	HB (g/dl)	PD/μl
Count (*n*)	102	99	112
Minimum *n*	0.5	4.4	40
Maximum *n*	35	13.6	184,600
Geometric mean (GM)	6.369	9.352	7715
Lower 95% CI of GM	5.095	8.967	5719
Upper 95% CI of GM	7.962	9.754	10,408

*Abbreviations*: *n* total number of samples analyzed, *HB (g/dl)* hemoglobin concentration, *PD/*μl parasite density

### Parasite genotyping

**Fig. 2:**
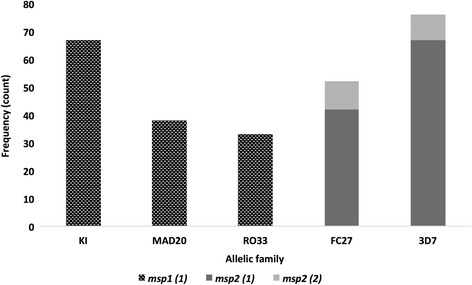
Distribution of *msp1* and *msp2* alleles. A graphical representation of data obtained from agarose gel electrophoresis of the allele-specific (*msp1* and *msp2* family) genotyping reactions. The number of samples that yielded one *msp1* allele (black checked) or one (dark grey) or two (light grey) *msp2* alleles in PCR reactions with the indicated allele-specific primer set are plotted

Out of the 115 samples collected 97 (84.3%) and 99 (86.1%) were successfully amplified for *msp1* and *msp2* genes, respectively. From the 97 samples with positive PCR products from the *msp1* PCR amplifications, a total of 126 PCR fragments were obtained. The overall frequency of the K1 allele was the highest, 67 (53.2%), followed by MAD20, 38 (30.1%), and RO33 at 33 (26.2%). Similarly, from the 99 samples that yielded positive PCR fragments for the *msp2* gene, a total of 52 samples contained parasites belonging to the FC27 allele family, of which 10 (19.2%) harbored double infections and thus resulted in a total of 62 PCR fragments (42 samples with single and 10 with double PCR fragments, 42.2%). Seventy-six samples contained parasites belonging to the 3D7 allele family, with 9/76 (11.8%) of the samples harboring two distinct parasite populations thereby producing a total frequency of 85 PCR fragments (57.8%) (Fig. 2). In total, 147 PCR fragments (62 FC alleles and 85 3D7 alleles) were obtained from the *msp2* targeted PCR amplifications from 99 samples.

### Multiplicity of infection

**Fig. 3:**
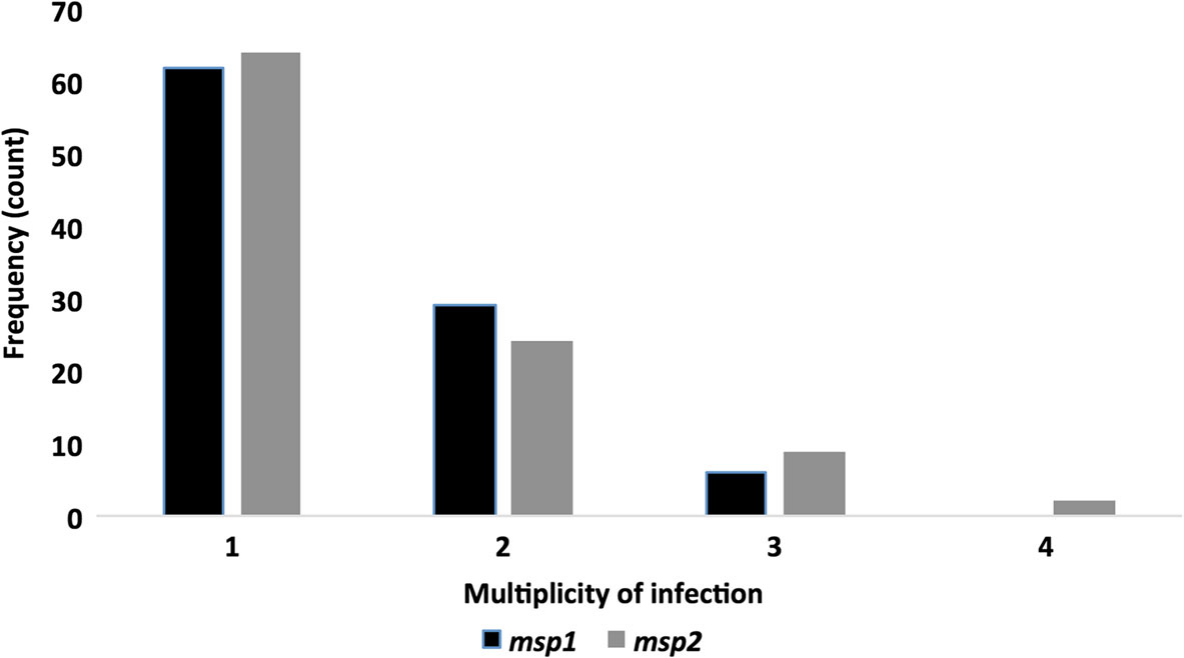
Multiplicity of infection for *msp1* and *msp2*. The number of samples are indicated that contained a total of one, two, three or four *msp1* alleles (black) or *msp2* alleles (light grey) when the results of all the genotyping reactions using the *msp1* K1, MAD20 or RO33 (black) or *msp2* FC27 or 3D7 (dark grey) allele-specific primer sets were combined

**Fig. 4:**
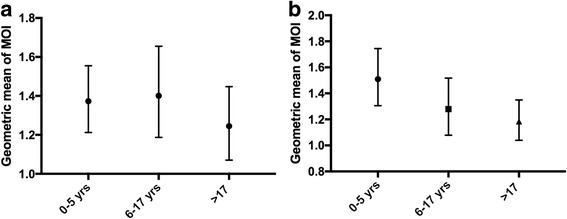
Geometric mean of MOI determined by *msp* 1 (**a**) and *msp* 2 (**b**) genotyping**.** Study participants were grouped into young children (0–5 yrs), older children (6–17 yrs) and adults (> 17 yrs). The geometric mean of the total number of PCR fragments obtained in each group per marker (*msp1* or *msp2*), which also represents the geometric mean of the MOI, is plotted with the 95% confidence interval

Combining the number of independent PCR fragments obtained for the three families, specific PCR amplifications for *msp1* revealed that 62 samples produced a single PCR fragment, two PCR fragments were obtained from 29 samples and 6 samples produced three different PCR fragments (Fig. 3). The GM for MOI obtained after *msp1* genotyping was 1.32 (95% CI: 1.22–1.42) (Additional file 1: Table S1). Combining the total number of PCR fragments obtained from a sample after *msp2* genotyping revealed that 64 samples contained a single PCR fragment, 24 samples contained two different fragments, 9 samples contained 3 PCR fragments and 2 samples contained 4 PCR fragments (Fig. 3). The GM for MOI obtained after *msp2* genotyping was 1.34 (95% CI: 1.24–1.46) (Additional file 1: Table S1). MOI determined by *msp2* genotyping was significantly negatively correlated with age (Spearman’s *r* = -0.2103, *P* = 0.044), but positively correlated with PD (Spearman’s *r* = 0.2290, *P* = 0.023). The GM of MOI determined by both *msp1* and *msp2* genotyping generally decreased with increasing age (Fig. 4a, b). The overall MOI in each sample was taken as the highest MOI predicted by either *msp1* or *msp2* genotyping. The GM for the overall MOI in this study population was 1.43 (95% CI: 1.32–1.55).

### IgG concentrations of GLURP(R0) and MSP3 antibodies

**Fig. 5:**
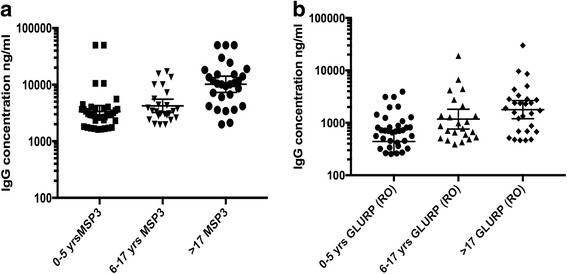
IgG concentrations of GLURP(R0) (**a**) MSP3 (**b**) and antibodies across the various age groups. A graphical represtation of the total IgG concentration (ng/ml) against GLURP(R0) (**a**) and MSP3 (**b**) in young children (0–5 yrs), older children (6–17 yrs) and adults (> 17 yrs). For each age group the GM with the 95% confidence interval of the antibody concentrations are indicated

IgG concentrations of antibodies against MSP3 were significantly higher than those against GLURP(R0) (Spearman’s *r* = 0.588, *P* < 0.0001). A significantly positive correlation was found to exist between age and IgG concentrations of both GLURP(R0) (Spearman’s *r* = 0.454, *P* < 0.0001) and MSP3 (Spearman’s *r* = 0.494, *P* < 0.0001) antibodies (Table 3, Fig. 5).

**Table 3:** Correlation between age and PD, R0 IgG and MSP3 IgG concentrations

	Age (yrs) *vs* PD	Age (yrs) *vs* RO	Age (yrs) *vs* MSP3
Spearman’s *r*	-0.09489	0.4546	0.4941
95% confidence interval	-0.2923 – 0.1103	0.2675 – 0.6087	0.3164 – 0.6384
*P*-value (two-tailed)	0.3501	< 0.0001	< 0.0001
Number of XY pairs	99	90	92

In contrast, PD was negatively correlated with MSP3 (Spearman’s *r* = -0.1944, *P* < 0.0502) as well as GLURP(R0) (Spearman’s *r* = -0.2461, *P* < 0.015) IgG concentrations. Seropositivity in this study was defined as serum antibody levels higher than the cutoff value, which is the mean of the antibody concentrations of the negative control plus 2 standard deviations. Applying the cutoff of 589 ng/μl and 3249 ng/μl for GLURP(R0) and MSP3, respectively; 67% of the participants were seropositive for GLURP(R0) and 63% for MSP3.

### Multiplicity of infection and GLURP(R0) and MSP3 antibody concentrations

Although the GM of the MOI in this study was relatively low, a significant association was identified with IgG concentrations of MSP3 (Independent-Samples Median Test, *P* = 0.008), but not GLURP(R0) antibodies (Independent-Samples Median Test, *P* = 0.053), across the varying *msp2* MOI categories. No significant association was found between either GLURP(R0) (Independent-Samples Median Test, *P* = 0.702) or MSP3 (Independent-Samples Median Test, *P* = 0.343) IgG concentrations and MOI determined by *msp1* genotyping.

## Discussion

This study accessed the genetic diversity and multiplicity of *P. falciparum* infection as well as antibody reponses to two asexual malaria vaccine candidate antigens (GLURP(R0) and MSP3) in a cohort of patients presenting to the hospital with uncomplicated malaria. The geometric mean (GM) of the parasite density (PD) in the study participants was 7715 parasites/μl (Additional file 1: Table S1). This was much lower than previously reported, (20,319/μl) for children with malaria in a different community within the same Region of Ghana [28]. Although there was a general decrease in PD with increasing age, no significant difference in PD among the study population was observed (Additional file 3: Figure S3) which is contrary to other reports, such as one from a study conducted in Kenya where young children (5–10 years) carried significantly higher parasite densities than older children (10–15 years) [30].

The most prevalent *msp1* allelic family was the KI allele (Fig. 2); this finding is consistent with our previous study [28] conducted along the coast of the Central Region of Ghana as well as other studies on asymptomatic and uncomplicated malaria patients in Cameroon [31] and Ethiopia [32]. However, this study identified the 3D7 allele of *msp2* as the most prevalent (Fig. 2), which was contrary to our previous study [28] but similar to other studies conducted in Ghana [33, 34].

The presence of concurrent *P. falciparum* parasite infections in this study population was low (Fig. 3), with a GM for MOI as 1.3 (Additional file 1: Table S1) indicating only a minority of patients being infected with multiple parasites. A previous study conducted in Southern Ghana in 2013 identified the GM for MOI of malaria patients as 1.3 [34]. However, our previous study conducted along the coast of the Central Region of Ghana reported a relatively higher geometric mean for MOI, 1.92 and 1.88 for MOI determined by *msp1* and *msp2*, respectively, in children with uncomplicated malaria [28].

There was no significant difference in MOI determined by either *msp1* or *msp2* genotyping among the three age groups studied (Fig. 4), which is consistent with recent reports from Sudan [35], Uganda [36] and Ethiopia [32]. Although there was a significant relationship between MOI and parasite density which was not observed in the Ethiopian study [32]. The MOI analysis might have been more sensitive if pyrosequencing [37] or capillary electrophoresis [38] had been used for the *msp1* and *msp2* genotyping as these techniques are able to distinguish between all possible different strains contained within a single PCR amplicon.

This study shows higher IgG levels against MSP3 than GLURP(R0) (Fig. 5), previously reported [39], however, IgG antibody levels for GLURP(R0) measured in children from Dodowa in the Greater Accra Region of Ghana in 2002 were higher than those for MSP3 [19]. This study reports increased IgG levels against GLURP(R0) and MSP3 with increasing age, which is similar to the previous report from Dodowa [19]. The overall seroprevalence of 67% for GLURP(R0) to 63% for MSP3 is also is similar to other studies which ranged from 58.6% [39] to 65% [40]. The results from this study also indicate that high antibody levels against GLURP(R0) and MSP3 are associated with lower parasite density [41–43] and likely protection from disease.

## Conclusions

The negative correlation between naturally occurring GLURP(R0) and MSP3 antibody levels and parasite density observed in this study suggest that augmenting the antibody response with the GMZ2 vaccine, or other vaccines that stimulates an increased production of antibodies against GLURP(R0) and MSP3, could enhance protection in the Central Region of Ghana. However, diversity in candidate vaccine antigens could influence the effect and will have to be monitored prior to and during vaccine trials.

## Additional files

Additional file 1: Table S1. Demographic features of the study participants per site and overall. *Abbreviations*: *n*, number of patients; GM, geometric mean; 95% CI, 95% confidence interval of the GM. Numbers in parenthesis represent the total number of patients. (DOCX 79 kb)

Additional file 2: Figure S1. Relationship between MSP3 (**a**) and GLURP(R0) (**b**) IgG seropositivity and Age (left y-axis) and PD (right y-axis). SPSS derived graphs showing the relationship between the mean Age (left y-axis) and PD/μl blood (right y-axis, log scale) of the study participants and IgG seropositivity to GLURP(R0) and MSP3. Error bars represent the standard error of the mean. 0, seronegative; 1, seropositive for MSP3 (**a**) and GLURP(R0) (**b**) antibodies. (DOCX 278 kb)

Additional file 3: Figure S2. A graphical representation of the correlation between log transformed PD and patient age (**a**) and stratified patient age (**b**). (DOCX 131 kb)

## Abbreviations

EDTA: Ethylenediaminetetraacetic acid
GLURP: Glutamate rich protein (*P. falciparum*)
GM: Geometric mean
MOI: Multiplicity of infection
MSP: Merozoite surface protein (*P. falciparum*)
PBS: Phosphate buffered saline
PD: Parasite density
