# Supplementary material for: Natural antibody responses to Plasmodium falciparum MSP3 and GLURP(R0) antigens are associated with low parasite densities in malaria patients living in the Central Region of Ghana
Source: Parasit Vectors. 2017 Aug 23;10:395. doi: 10.1186/s13071-017-2338-7 (PMC5569498; doi:10.1186/s13071-017-2338-7)
Supplement: Supplementary file 1 — Demographic features of the study participants per site and overall. Abbreviations: n, number of patients; GM, geometric mean; 95% CI, 95% confidence interval of the GM. Numbers in parenthesis represent the total number of patients. (DOCX 79 kb) [file 13071_2017_2338_MOESM1_ESM.docx]

Additional file 1: Table S1

Demographic features of the study participants per site and overall.

| Site |  | Age | PD/μl | HB / g/dL | *msp1* MOI | *msp2* MOI |
| --- | --- | --- | --- | --- | --- | --- |
| Abura (32) | N | 24 | 32 | 22 | 28 | 30 |
|  | Mean | 8.64 | 29241 | 9.8 | 1.39 | 1.57 |
|  | Geometric Mean | 5.81 | 6446 | 9.6 | 1.29 | 1.37 |
|  | Lower 95% CI of GM | 3.94 | 3240 | 8.9 | 1.12 | 1.16 |
|  | Higher 95% CI of GM | 8.52 | 12515 | 10.4 | 1.48 | 1.62 |
| Assin Fosu (47) | N | 47 | 47 | 46 | 39 | 38 |
|  | Mean | 11.66 | 16033 | 9.8 | 1.44 | 1.53 |
|  | Geometric Mean | 7.48 | 9674 | 9.6 | 1.32 | 1.41 |
|  | Lower 95% CI of GM | 5.53 | 6790 | 9.0 | 1.18 | 1.25 |
|  | Higher 95% CI of GM | 10.07 | 13451 | 10.2 | 1.48 | 1.59 |
| Twifo Praso (36) | N | 31 | 33 | 31 | 30 | 31 |
|  | Mean | 10.50 | 17755 | 9.0 | 1.43 | 1.35 |
|  | Geometric Mean | 5.36 | 6206 | 8.8 | 1.34 | 1.24 |
|  | Lower 95% CI of GM | 3.34 | 3362 | 8.1 | 1.19 | 1.10 |
|  | Higher 95% CI of GM | 8.72 | 10976 | 9.4 | 1.52 | 1.44 |
| Total (115) | N | 102 | 112 | 99 | 97 | 99 |
|  | Mean | 10.60 | 20533 | 9.5 | 1.42 | 1.49 |
|  | Geometric Mean | 6.37 | 7715 | 9.4 | 1.32 | 1.34 |
|  | Lower 95% CI of GM | 5.10 | 5719 | 9.0 | 1.22 | 1.24 |
|  | Higher 95% CI of GM | 7.96 | 10408 | 9.8 | 1.42 | 1.46 |

*Abbreviations*: *n*, number of patients; GM, geometric mean; 95% CI, 95% confidence interval of the GM. Numbers in parenthesis represent the total number of patients
