# Supplementary material for: Natural antibody responses to Plasmodium falciparum MSP3 and GLURP(R0) antigens are associated with low parasite densities in malaria patients living in the Central Region of Ghana
Source: Parasit Vectors. 2017 Aug 23;10:395. doi: 10.1186/s13071-017-2338-7 (PMC5569498; doi:10.1186/s13071-017-2338-7)
Supplement: Supplementary file 2 — Relationship between MSP3 (a) and GLURP(R0) (b) IgG seropositivity and Age (left y-axis) and PD (right y-axis). SPSS derived graphs showing the relationship between the mean Age (left y-axis) and PD/μl blood (right y-axis, log scale) of the study participants and IgG seropositivity to GLURP(R0) and MSP3. Error bars represent the standard error of the mean. 0, seronegative; 1, seropositive for MSP3 (a) and GLURP(R0) (b) antibodies. (DOCX 278 kb) [file 13071_2017_2338_MOESM2_ESM.docx]

Additional file 2: Relationship between MSP3 (a) and GLURP(R0) (b) IgG seropositivity and Age (left y-axis) and PD ( right y-axis)

a

b


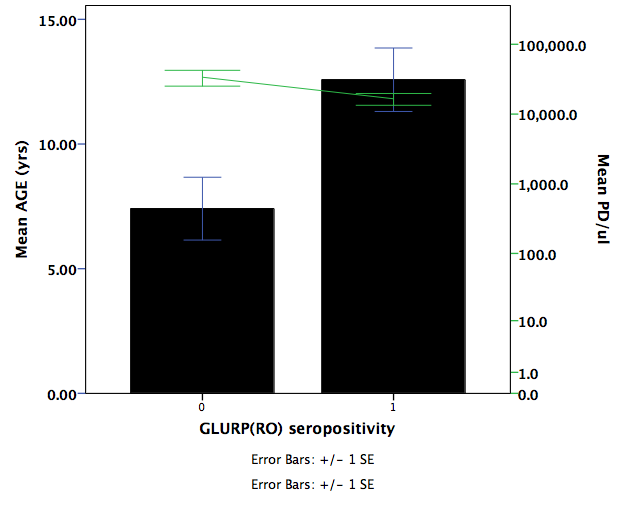


SPSS derived graphs showing the relationship between the mean mean Age (left y-axis) and PD/μl blood (right y-axis, log scale) of the study participants and IgG seropositivity to GLURP(R0) and MSP3. Error bars represent the standard error of the mean. 0, seronegative; 1, seropositive for MSP3 (a) and GLURP(R0) (b) antibodies.
