# Supplementary material for: Natural antibody responses to Plasmodium falciparum MSP3 and GLURP(R0) antigens are associated with low parasite densities in malaria patients living in the Central Region of Ghana
Source: Parasit Vectors. 2017 Aug 23;10:395. doi: 10.1186/s13071-017-2338-7 (PMC5569498; doi:10.1186/s13071-017-2338-7)
Supplement: Supplementary file 3 — A graphical representation of the correlation between log transformed PD and patient age (a) and stratified patient age (b). (DOCX 131 kb) [file 13071_2017_2338_MOESM3_ESM.docx]

Additional file 3: A graphical representation of the correlation between log transformed PD and patient age (a) and stratified patient age (b)

b

a
